# Effects of Dihydrophaseic Acid 3′-*O*-β-d-Glucopyranoside Isolated from *Lycii radicis* Cortex on Osteoblast Differentiation

**DOI:** 10.3390/molecules21091260

**Published:** 2016-09-21

**Authors:** Eunkuk Park, Mun-Chang Kim, Chun Whan Choi, Jeonghyun Kim, Hyun-Seok Jin, Ryunjin Lee, Ji-Won Lee, Jin-Hyok Park, Dam Huh, Seon-Yong Jeong

**Affiliations:** 1 Department of Medical Genetics, Ajou University School of Medicine, Suwon 16499, Korea; jude0815@hotmail.com (E.P.); monotos@hanmail.net (M.-C.K.); danbi37kjh@hanmail.net (J.K.); ryun2@ajou.ac.kr (R.L.); 2 Department of Biomedical Sciences, Ajou University Graduate School of Medicine, Suwon 16499, Korea; 3 Bio-Center, Gyeonggi Institute of Science & Technology Promotion, Suwon 16229, Korea; cwchoi78@gmail.com; 4 Department of Biomedical Laboratory Science, College of Life and Health Sciences, Hoseo University, Asan 31499, Korea; microchin@hanmail.net; 5 Korea Food Research Institute, Seongnam 13539, Korea; dnjs0004@naver.com; 6 Dongwoodang Pharmacy Co., Ltd., Yeongchen 38819, Korea; navy9376@hanmail.net

**Keywords:** osteoporosis, herbal medicine, bioactive compound, dihydrophaseic acid 3′-*O*-β-d-glucopyranoside, osteoblast, osteoclast, bone remodeling

## Abstract

Our previous study showed that ethanol extract of *Lycii* *radicis* cortex (LRC) prevented the loss of bone mineral density in ovariectomized mice by promoting the differentiation of osteoblast linage cells. Here, we performed fractionation and isolation of the bioactive compound(s) responsible for the bone formation–enhancing effect of LRC extract. A known sesquiterpene glucoside, (1′*R*,3′*S*,5′*R*,8′*S*,2*Z*,4*E*)-dihydrophaseic acid 3′-*O*-β-d-glucopyranoside (abbreviated as DPA3G), was isolated from LRC extract and identified as a candidate constituent. We investigated the effects of DPA3G on osteoblast and osteoclast differentiation, which play fundamental roles in bone formation and bone resorption, respectively, during bone remodeling. The DPA3G fraction treatment in mesenchymal stem cell line C3H10T1/2 and preosteoblast cell line MC3T3-E1 significantly enhanced cell proliferation and alkaline phosphatase activity in both cell lines compared to the untreated control cells. Furthermore, DPA3G significantly increased mineralized nodule formation and the mRNA expression of osteoblastogenesis markers, *Alpl*, *Runx2*, and *Bglap*, in MC3T3-E1 cells. The DPA3G treatment, however, did not influence osteoclast differentiation in primary-cultured monocytes of mouse bone marrow. Because osteoblastic and osteoclastic precursor cells coexist in vivo, we tested the DPA3G effects under the co-culture condition of MC3T3-E1 cells and monocytes. Remarkably, DPA3G enhanced not only osteoblast differentiation of MC3T3-El cells but also osteoclast differentiation of monocytes, indicating that DPA3G plays a role in the maintenance of the normal bone remodeling balance. Our results suggest that DPA3G may be a good candidate for the treatment of osteoporosis.

## 1. Introduction

Bone is dynamic tissue that undergoes continuous remodeling with bone formation and resorption to maintain homeostasis in the healthy skeleton [1,2]. Bone remodeling occurs through repeated cycles of reshaping or replacement of bone. Through natural processes, aged and damaged bone cells are removed every day, and equal amounts of new mineral deposition are newly formed, resulting in the gradual restructuring of bone. Bone remodeling requires the coordinated action of several types of bone cells, namely bone-lining cells, osteocytes, osteoclasts, and osteoblasts [1,2]. Osteoblasts, the bone-forming cells, are differentiated from mesenchymal stem cells (MSCs); they then differentiate into osteocytes, which play a fundamental role in the initiation of bone remodeling. Osteoclasts, the bone-resorbing cells, are differentiated from mononuclear cells of the monocyte/macrophage lineage [1,2]. Bone formation involves osteoblast proliferation, differentiation with alkaline phosphatase activation, collagen synthesis, and mineralization [3]. Bone resorption involves osteoclast differentiation with tartrate-resistant acid phosphatase activation [4,5]. Coupling between bone formation and bone resorption involves the interaction of wide range of cell types in a basic multicellular unit during bone remodeling [6,7]. Osteoblast and osteoclast lineage precursor cells communicate with each other through cell–cell contact via gap junctions; diffusible paracrine factors, such as growth factors, cytokines, chemokines, and so on; and cell–bone matrix interaction [6]. Bone resorption is needed to replace old or damaged bone, and therefore, osteoclast differentiation is the initiation step of bone remodeling. Monocytic precursors’ differentiation into mature osteoclasts on the bone surface depends on diffusible paracrine factors produced by osteoblast-lineage cells, particularly macrophage colony-stimulating factor (M-CSF) and receptor activator of nuclear factor kappa-B ligand (RANKL), indicating that osteoblast differentiation is essential for the initiation of osteoclast differentiation in vivo [5,8]. In contrast, monocytes contribute to promote the osteoblast formation from MSCs [6,7,9].

Imbalanced regulation of the bone-remodeling process results in metabolic bone diseases, such as osteoporosis and osteopenia [1]. Osteoporosis is a common disease characterized by a systemic impairment of bone mass and microarchitecture [10,11]. In osteoporosis patients, particularly postmenopausal women, excessive bone resorption occurs compared to bone rebuilding, which leads to an enhanced risk of bone fragility and susceptibility to fractures [12]. Currently, the mainstay pharmacological treatment for osteoporosis is focused on inhibitors of bone resorption, such as bisphosphonates, or stimulators of bone formation, such as parathyroid hormone analogs. Although a number of effective drugs are currently available for the treatment of osteoporosis, there are still limitations, including side effects and unmet needs [13,14,15].

Herbal medicine is a term that is widely used to describe alternative therapies or combination treatments with modern medicines for many diseases [16,17,18]. Discovering the pharmacologically active compounds from natural products has been a useful strategy for drug discovery and design [16,19]. For the treatment of bone-related diseases, such as osteoporosis, many Chinese herbal medicines have a long tradition of use, and their bioactive compounds displaying osteoprotective and related properties have been identified [20,21,22,23]. Because long-term treatment is required for osteoporosis, herbal medicine has been thought to be a good strategy for alternative treatment of osteoporosis with fewer negative effects. Our previous study suggested that a natural herbal medicine, *Lycii radicis* cortex (LRC) extract, may be a good candidate as an alternative long-term treatment for osteoporosis without negative effects [24]. LRC, which is *Lycium chinense* root bark, is extensively used in East Asia as a traditional medicine [25,26]. We previously reported that LRC extract prevented loss of bone mineral density in ovariectomized mice [24]. The in vitro study revealed that the LRC extract promotes the differentiation of osteoblast linage cells rather than the inhibition of osteoclastic differentiation [24]. However, another study reported that LRC inhibited RANKL-induced osteoclast differentiation via the suppression of osteoclastogenesis-related markers [27]. Therefore, to clarify the mechanisms by which the LRC extract affects bone formation and/or resorption, it is necessary to perform experiments under more physiological in vitro conditions, where osteoblastic and osteoclastic precursor cells coexist.

In this study, we aimed to identify the bioactive compound(s) responsible for the bone formation–enhancing effect of LRC extract. We carried out fractionation and isolation of LRC extract and found an uncommon, known single compound with an unknown biological or pharmacological function. Next, we evaluated the effects of the identified compound on osteoblastic and osteoclastic precursor cell differentiation in single-culture and co-culture of preosteoblasts and primary monocytes. 

## 2. Results and Discussion

### 2.1. Isolation and Identification of DPA3G as a Bioactive Component of the LRC Extract for Enhancing Osteoblast Differentiation

**Figure 1:**
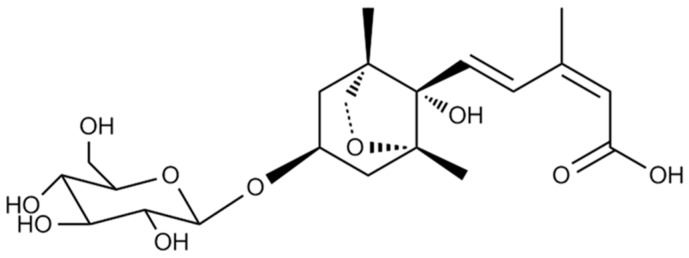
Chemical structure of the isolated (1′*R*,2′*S*,5′*R*,8′*S*,2′*Z*,4′*E*)-dihydrophaseic acid 3′-*O*-β-d-glucopyranoside (DPA3G).

Our previous study demonstrated that ethanol extract of LRC enhanced osteoblast differentiation in MC3T3-E1 preosteoblast cells and prevented the loss of bone mineral density in ovariectomized mice [24]. Several studies have demonstrated that LRC extract contains a variety of physiologically active compounds [25,26]. We also identified the 13 most abundant constituents, including lyciumoside III, lyciumin A, and lyciumin B from the LRC ethanol extract using a high-performance liquid chromatography (HPLC)–electrospray ionization (ESI)–tandem mass spectrometry system [24]. To identify the bioactive compound(s) responsible for the bone formation–enhancing effect of LRC extract, we conducted fractionation of the 70% ethanol extract of LRC. The extract was fractionated into dichloromethane, ethyl acetate, *n*-butanol, and aqueous fractions, and the aqueous fraction (D) was further fractionated (Figure S1). An alkaline phosphatase (ALP) activity assay of each fraction in preosteoblast MC3T3-E1 cells led to the isolation of bioactive fractions. The constituent of the final active subfraction (D2-3-4-2) was analyzed by proton nuclear magnetic resonance (^1^H-NMR), carbon-13 nuclear magnetic resonance (^13^C-NMR), and mass spectrometry analyses (Figure S2). As a result, the known sesquiterpene glucoside (1′*R*,3′*S*,5′*R*,8′*S*,2*Z*,4*E*)-dihydrophaseic acid 3′-*O*-β-d-glucopyranoside (abbreviated as DPA3G), was identified (Figure 1). The molecular formula of DPA3G is C_21_H_32_O_10_.

DPA3G was previously isolated from lotus (*Nelumbo nucifera* Gaertner, Nymphaeaceae) and jujube (*Zizyphus jujube* var. *spinose*) [28,29]. The biological function of DPA3G has not yet been determined, but its stereoisomer (1′*R*,3′*S*,5′*R*,8′*S*,2*E*,4*E*)-dihydrophaseic acid 3′-*O*-β-d-gluco-pyranoside isolated from the stem bark of *Ginkgo biloba* was reported to have anti-inflammatory effects via the inhibition of tumor necrosis factor-alpha (TNFα)-induced nuclear factor kappa-B (NF-κB) transcriptional activity and a transactivational effect of peroxisome proliferator-activated receptor-β/δ (PPARβ/δ) [30]. A previous study reported that NF-κB reduced Runx2 and β-catenin binding to osteocalcin and bone sialoprotein promoters and that NF-κB inhibition in osteoblasts increased osteocalcin expression in mice with periodontal disease [31]. Further, inhibition of NF-κB by orthosilicic acid treatment resulted in activation of Runx2, the master transcription factor for osteoblast precursor differentiation, and suppression of NFATc1 expression, the key transcription gene for osteoclast precursor differentiation [32]. PPARβ/δ is known to govern Wnt signaling and bone turnover [33]. Activation of PPARβ/δ by agonist treatment in ovariectomized osteoporotic mice led to rebalancing of bone turnover and restoration of normal bone density [33]. These results suggest that DPA3G is one of the candidate constituents responsible for the anti-osteoporotic effect of LRC extract and may function via NF-κB inhibition and/or PPARβ/δ activation.

### 2.2. DPA3G Increased the Cellular Proliferation, Differentiation, and Mineralized Nodule Formation of Osteoblasts

There are three stages of osteoblast differentiation: cell proliferation, matrix maturation, and matrix mineralization [34]. The orthodox methods for evaluating osteoblast differentiation include cellular proliferation, ALP activity, mineralization, and mRNA expression of osteoblast differentiation markers, such as *Alpl* (ALP), *Runx2* (runt-related transcription factor 2, Runx2), and *Bglap* (bone gamma carboxyglutamate protein, osteocalcin) genes [35,36,37,38].

**Figure 2:**
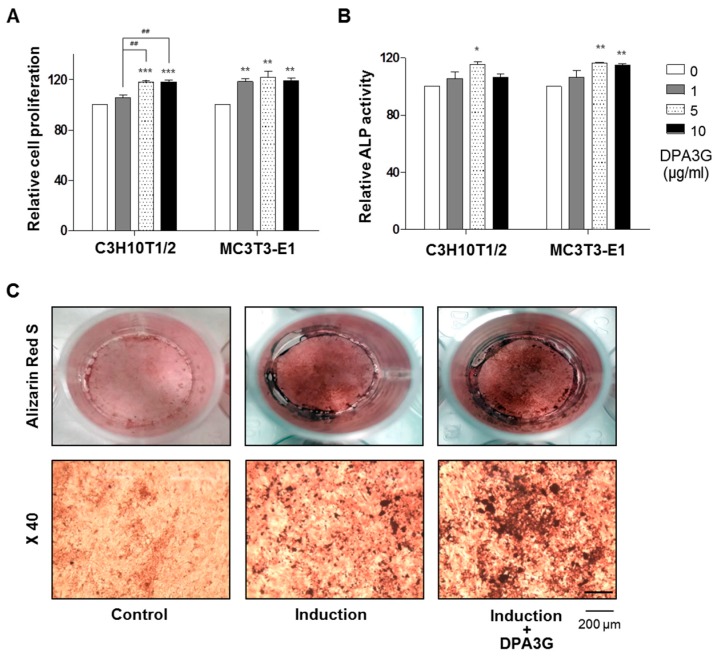
Effects of (1′*R*,2′*S*,5′*R*,8′*S*,2′*Z*,4′*E*)-dihydrophaseic acid 3′-*O*-β-d-glucopyranoside (DPA3G) on cellular proliferation, differentiation, and mineralized nodule formation of the osteoblast-lineage cell lines. (**A**) Assessment of the cellular proliferation in the DPA3G fraction–treated C3H10T1/2 and MC3T3-E1 cells. Cells were treated with three different concentrations of DPA3G fraction (1, 5, and 10 µg/mL) for 3 days, and then cell viability was assessed. **, ***: *p* < 0.01, *p* < 0.001 vs. 0, and ^##^: *p* < 0.01 vs. 1 (Tukey’s HSD post hoc test, ANOVA); (**B**) Assessment of the alkaline phosphatase (ALP) activity in the DPA3G fraction–treated C3H10T1/2 and MC3T3-E1 cells. After induction of osteoblast differentiation, cells were treated with three different concentrations of DPA3G fraction (1, 5, and 10 µg/mL) for 3 days, and then ALP activity was assessed. *, **: *p* < 0.05, *p* < 0.01 vs. 0 (Tukey’s HSD post hoc test, ANOVA). (**C**) Assessment of in vitro bone mineralization in the DPA3G fraction–treated MC3T3-E1 cells. After induction of osteoblast differentiation, cells were treated with 5 µg/mL of DPA3G fraction for 21 days, and then cells were stained with alizarin red S. The positively stained nodules were visualized under a microscope at magnifications of 1 and 40. Control: non-induction of osteoblast differentiation. Induction: induction of osteoblast differentiation with 50 μg/mL of ascorbic acid and 10 mM of β-glycerophosphate. All of the experiments were repeated three times.

To confirm the bioactivity of the isolated DPA3G in osteoblasts, three different concentrations (1, 5, and 10 μg/mL) of the fraction containing DPA3G were treated in the osteoblast-lineage C3H10T1/2 and MC3T3-E1 cells, and the proliferation and ALP activity of the cells were examined (Figure 2). After 3 days of incubation, the cell viability and active bone formation were determined by water-soluble tetrazolium salt (WST) and ALP assays, respectively. DPA3G significantly enhanced cellular proliferation in both cell lines (Figure 2A). The highest ALP activity was observed with the 5 μg/mL DPA3G treatment in both cell lines (Figure 2B). ALP, a glycoprotein found on the surface of osteoblasts, increases during active bone formation with the induction of osteoblast activity; thus, ALP plays a crucial role in the mineralization of newly formed bone [39,40].

Next, we examined whether DPA3G stimulated mineralized nodule formation in MC3T3-E1 cells. Most bone matrix is mineralized by osteoblasts, resulting in the production of calcium and phosphate-based minerals; these induce the mineralization of bone and many matrix proteins [41]. As mineralized matrix and nodule formation are key factors in the development of bone formation [41,42], Alizarin Red S staining is a common histochemical method for the measurement of calcium deposits in mineralized osteoblast cells [43]. Positive Alizarin Red S staining signifies the presence of calcium phosphate and osteoblast mineralization, indicating successful in vitro bone formation. After osteoblast induction, the DPA3G fraction (5 μg/mL) was treated in MC3T3-E1 cells for 21 days. Markedly increased mineralized nodule formation was observed in DPA3G-treated cells compared to the untreated cells (Figure 2C).

**Figure 3:**
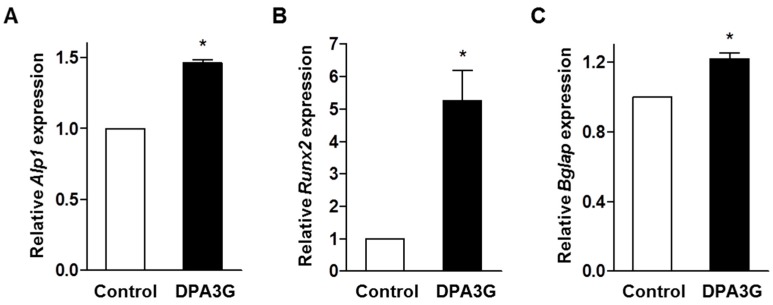
Effects of (1′*R*,2′*S*,5′*R*,8′*S*,2′*Z*,4ʹ*E*)-dihydrophaseic acid 3′-*O*-β-d-glucopyranoside (DPA3G) on the mRNA expression levels of osteoblast differentiation markers in preosteoblast MC3T3-E1 cells. After induction of osteoblast differentiation, cells were treated with 5 µg/mL of DPA3G fraction for 3 days and then total RNA of the cells was extracted. The mRNA expression levels of *Alpl* (alkaline phosphatase, ALP) (**A**); *Runx2* (runt-related transcription factor 2, Runx2) (**B**); and *Bglap* (bone gamma carboxyglutamate protein, Osteocalcin) (**C**) genes were assessed by quantitative reverse-transcription polymerase chain reaction (qRT-PCR) and then normalized to *Gapdh* (glyceraldehyde 3-phosphate dehydrogenase) mRNA expression. Control: non-DPA3G-treated cells. *: *p* < 0.05 vs. Control (Student’s *t*-test). All of the experiments were repeated three times.

We examined the effect of DPA3G on the expression of bone remodeling markers *Alpl*, *Runx2*, and *Bglap* (osteocalcin). MC3T3-E1 cells were treated with DPA3G fraction (5 μg/mL) for 3 days. The mRNA expression levels of *Alpl*, *Runx2*, and *Bglap* were measured by quantitative RT-PCR (qRT-PCR). The expression levels of *Alpl*, *Runx2*, and *Bglap* were significantly increased in the DPA3G-treated cells compared to the untreated control cells (Figure 3). ALP is a central enzyme in the mineralization of newly formed bone [40], and Runx2 is essential for osteoblast differentiation, stimulating the main bone matrix proteins during the early stages of osteoblast differentiation [44]. Differentiated osteoblasts express high levels of Osteocalcin correlated with increases in bone mineral density [45,46].

The above results indicate that DPA3G enhances the proliferation, differentiation, and mineralized nodule formation of bone–forming osteoblasts. Although the exact molecular mechanisms of pharmacological action of DPA3G on these effects remain unclear, based on the stereoisomer’s function [30], DPA3G seems to play a role in the stimulation of osteoblastogenesis via NF-κB inhibition and/or PPARβ/δ activation

### 2.3. DPA3G Did Not Influence Differentiation of Osteoclasts

**Figure 4:**
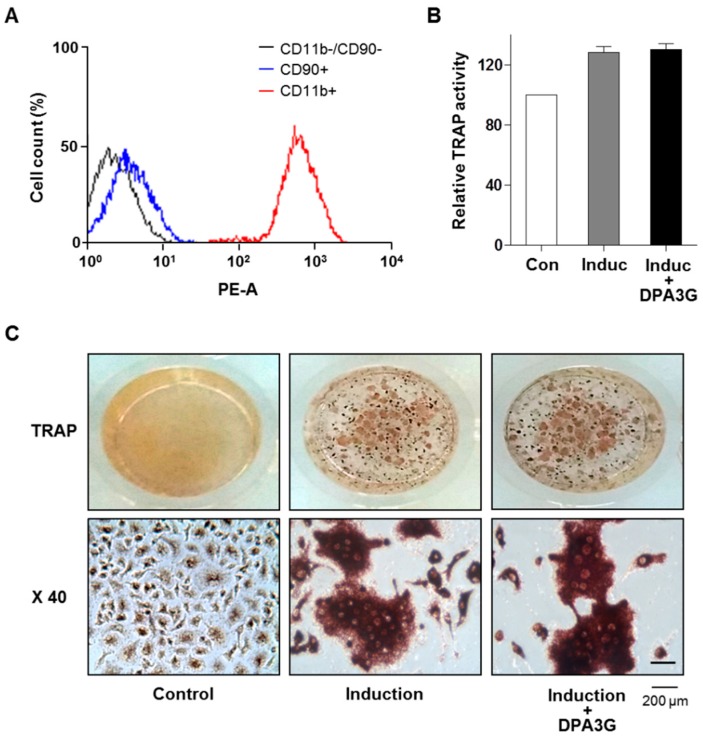
Effects of (1′*R*,2′*S*,5′*R*,8′*S*,2′*Z*,4′*E*)-dihydrophaseic acid 3′-*O*-β-d-glucopyranoside (DPA3G) on osteoclast differentiation of primary-cultured monocytes. (**A**) Validation of successful isolation of monocytes from mouse bone marrow. Primary-cultured monocytes were identified by immunophenotypic analysis with a monocyte-specific surface positive marker (PE-conjugated CD11b antibody). The absence of contamination of mesenchymal stem cells (MSCs) was confirmed by an immunophenotypic analysis with an MSC-positive marker (PE-conjugated CD90 antibody) using fluorescence-activated cell sorting (FACS) analysis; (**B**,**C**) Assessment of tartrate-resistant acid phosphatase (TRAP) activity in the DPA3G fraction-treated monocyte cells. After induction of osteoclast differentiation, cells were treated with 5 µg/mL of DPA3G fraction for 5 days, and then TRAP activity was assessed (**B**). The cells were also stained with a TRAP staining kit, and the differentiated osteoclast cells were visualized under a microscope at magnifications of 1 and 40 (**C**). Control: non-induction of osteoclast differentiation. Induction: induction of osteoclast differentiation with 30 ng/mL of macrophage colony-stimulating factor (M-CSF) and 50 ng/mL of receptor activator of nuclear factor kappa-B ligand (RANKL). All of the experiments were repeated three times.

We also investigated the effects of DPA3G on the differentiation of osteoclasts. Monocytic precursors differentiate into mature osteoclasts, and osteoclasts function through the degradation and removal of both the inorganic mineral and organic matrix [5]. Monocytes were isolated from bone marrow of 6-week-old mice, and their successful isolation and culture were confirmed by fluorescence-activated cell sorting (FACS) analysis with monocyte-specific surface markers (anti-CD11b antibody; Figure 4A). Differentiation of primary-cultured monocytes to osteoclasts was induced by treatment of M-CSF and RANKL [5,8]. After induction of osteoclast differentiation, DPA3G fraction (5 μg/mL) was treated in the primary monocytes for 5 days. Tartrate-resistant acid phosphatase (TRAP) activity assay and TRAP staining results showed no difference between DPA3G-treated and untreated primary monocytes (Figure 4B,C), indicating that DPA3G plays a role in stimulation of osteoblast differentiation rather than inhibition of osteoclast differentiation.

### 2.4. DPA3G Enhanced both Osteoblast and Osteoclast Differentiation in the MC3T3-E1 and Primary Monocyte Co-Culture System

**Figure 5:**
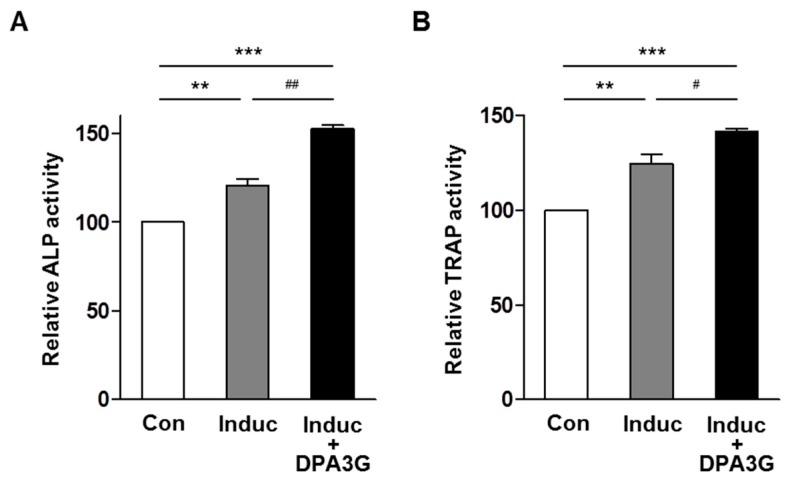
Effects of (1′*R*,2′*S*,5′*R*,8′*S*,2′*Z*,4′*E*)-dihydrophaseic acid 3′-*O*-β-d-glucopyranoside (DPA3G) on osteoblast and osteoclast differentiation in the co-culture of preosteoblasts and primary monocytes. Co-cultured MC3T3-E1 and primary monocyte cells were treated with osteoblast differentiation reagents, ascorbic acid, and β-glycerophosphate, and then co-treated with 5 µg/mL of DPA3G fraction for 5 days. Alkaline phosphatase (ALP) activity (**A**) and tartrate-resistant acid phosphatase (TRAP) activity (**B**) were assessed in the co-culture cells. Con: non-induction control of osteoblast differentiation. Induc: induction of osteoblast differentiation with 50 μg/mL of ascorbic acid and 10 mM of β-glycerophosphate. **, ***: *p* < 0.01, *p* < 0.001 vs. Control, and ^#^, ^##^: *p* < 0.05, *p* < 0.01 vs. Induction (Tukey’s HSD post hoc test, ANOVA). All of the experiments were repeated three times.

In the skeletal system, homeostasis of bone remodeling is maintained by the balance of bone resorption and bone formation [1,2]. Finally, we investigated the effects of DPA3G on bone formation and bone resorption under the more physiological conditions in vitro. We established the co-culture system of osteoblast precursor MC3T3-E1 cells and osteoclast precursor monocyte cells based on previous co-culture studies [47,48,49]. After induction of osteoblast differentiation with ascorbic acid and β-glycerophosphate, the effects of DPA3G on ALP activity for osteoblast differentiation and TRAP activity for osteoclast differentiation were examined. Through osteoblast differentiation induction, both ALP and TRAP activities were increased (Figure 5), and the ALP and TRAP activities of the tested co-cultured cells were significantly higher than those of the separately cultured MC3T3-E1 and monocyte cells, respectively (Figure S3), indicating the proper functioning of the co-culture system. Remarkably, treatment of DPA3G fraction (5 μg/mL) significantly enhanced not only ALP activity but also TRAP activity compared with untreated cells (Figure 5).

Because DPA3G did not affect osteoclast differentiation in single-culture of monocytes (Figure 4), the enhancement of osteoclast differentiation by DPA3G treatment in co-culture may be due to the increased M-CSF and RANKL in the co-culture media, which resulted from the enhanced osteoblast differentiation by DPA3G treatment. To confirm this, mRNA expression levels of *Tnfs11* (tumor necrosis factor superfamily member 11, RANKL) gene were compared between DPA3G-treated and untreated co-culture cells. As expected, the DPA3G treatment significantly increased expression of *Tnfs11* (Figure S4). All the results of co-culture experiments indicate that DPA3G may contribute to enhanced coupling between osteoblasts and osteoclasts in a paracrine fashion.

Further research using ovariectomized mice treated with DPA3G will help elucidate how DPA3G is responsible for the bone formation–enhancing effect of the LRC extract in vivo. However, because a large amount of DPA3G is required for an in vivo experiment and DPA3G is not commercially available, we could not conduct an in vivo experiment using DPA3G. Establishment of a mass production system of DPA3G from the LRC extract is needed to investigate the function of DPA3G in vivo.

## 3. Experimental Section

### 3.1. Fractionation, Isolation, and Structure Elucidation of the Bioactive Component

Seventy percent ethanol extract of LRC (254 g) was evaporated, suspended in H_2_O, and then partitioned successively with the appropriate solvents to give dichloromethane (A, 1.6 g), ethyl acetate (B, 3.6 g), *n*-butanol (C, 120.1 g), and aqueous (D, 128.1 g) fractions (Figure S1). The activity of osteoblast differentiation was evaluated in each fraction. Bioactivity-guided fractionations are indicated in Figure S1. Fraction D was chromatographed using Diaion HP-20 gel (1000 g) column chromatography, eluted with a gradient H_2_O-methanol (MeOH) solvent system (O% MeOH, 35% MeOH, 70% MeOH, and 100% MeOH) to give four fractions (D1–D4). Fraction D2 (14.5 g) was subjected to RP-18 gel (200 g) column chromatography eluted with H_2_O-MeOH (100:0 to 0:100) to afford seven subfractions (D2-1 to D2-7). Subfraction D2-3 (724.8 mg) was subjected to preparative high-performance liquid chromatography (HPLC) eluted with H_2_O–MeOH (100:0 to 0:100) to afford five subfractions (D2-3-1 to D2-3-5). Subfraction D2-3-4 (63.4 mg) was subjected to preparative HPLC eluted with MeOH-H_2_O/0.1% formic acid (10:90) to afford two subfractions (D2-3-4-1 and D2-3-4-2). Because subfraction D2-3-4-2 significantly enhanced ALP activity in both cell lines, C3H10T1/2 MSCs and MC3T3-E1 preosteoblasts (Figure 2B), this subfraction was deduced to be a “bioactive fraction”. 

The structure of the compound in the bioactive fraction was elucidated by proton nuclear magnetic resonance (^1^H-NMR), carbon-13 nuclear magnetic resonance (^13^C-NMR), and mass spectrometry analyses (Figure S2), as well as by comparison with the previously reported data [29]. ^1^H (700 MHz) and ^13^C (175 MHz) NMR spectra were recorded on an Ascend 700 MHz NMR spectrometer (Bruker, Billerica, MA, USA) in MeOH-δ^4^ at 25 °C; chemical shifts are given in values (ppm) based on those of the solvent signals (^1^H 3.31 and ^13^C 49.0 ppm). An electrospray ionization (ESI)–tandem mass spectrometry analysis was performed using the Accela liquid chromatographic system (Thermo Fisher Scientific, Waltham, MA, USA) coupled with the LTQ-Orbitrap XL mass spectrometer (Thermo Fisher Scientific). The data were collected and analyzed using the Thermo Fisher Xcalibur software package (version 2.2, Thermo Fisher Scientific, San Jose, CA, USA). The mass spectrometer equipped with an ESI source was operated in negative ionization mode using the following operating parameters: an electrospray voltage of 4.0 kV, a sheath gas flow rate of 30 arbitrary units, an auxiliary gas flow rate of 8 arbitrary units, a capillary temperature of 275 °C, and a capillary voltage of 30 V. Instrument calibration was performed externally prior to each sequence using a calibration solution. Nitrogen (99.95%) was used as a sheath gas and as an auxiliary gas. The nitrogen served as a collision gas in the high-energy collisional dissociation cell and as a bath gas in the C-trap.

### 3.2. Cell Culture

Cells from the murine mesenchymal progenitor cell line C3H10T1/2 were purchased from the Korean Cell Line Bank (Seoul, Korea) and grown in Dulbecco’s Modified Eagle’s (DMEM) medium supplemented with 10% fetal bovine serum (FBS) (Sigma-Aldrich, St. Louis, MO, USA), 100 U/mL of penicillin (Duchefa; RV Haarlem, The Netherlands), and 100 μg/mL of streptomycin (Duchefa). Cells from the murine preosteoblast cell line MC3T3-E1 were purchased from the RIKEN Cell Bank (Tsukuba, Japan) and cultured in α-modified minimal essential medium (α-MEM) supplemented with 10% FBS, penicillin (100 U/mL), and streptomycin (100 μg/mL). All cultured cells were incubated in a humidified atmosphere at 37 °C and 5% CO_2._ The cells were used at passages 5–10 after purchase for all experiments.

To prepare primary-cultured monocytes, the bone marrow of femoral bones of 6-week-old mice was removed by flushing with a fine-bore syringe into α-MEM medium in the presence of 30 ng/mL of macrophage colony-stimulating factor (M-CSF) (PeproTech, Rocky Hill, NJ, USA) for 3–5 days [5,8]. The isolated monocytes were validated by immunophenotypic analysis with a CD11b antibody (BioLegend, San Diego, CA, USA) using the FACS Aria III Cell Sorter (BD Biosciences, San Jose, CA, USA) and FACS Diva software (BD Biosciences). The animal research procedures were approved by the Animal Care and Use Committee of the Ajou University School of Medicine (IACUC No. 2014-0066), and all experiments were conducted in accordance with the institutional guidelines established by the committee. All efforts were made to minimize animal suffering and to reduce the number of mice used.

### 3.3. Water-soluble Tetrazolium Salt (WST) Assay in Osteoblast Cells

The osteoblast-lineage C3H10T1/2 and MC3T3-E1 cells (3 × 10^3^ cells/well) were incubated in a 96-well plate overnight and treated with different concentrations of DPA3G fraction (1, 5, and 10 μg/mL) for 3 days. The treatment dose of DPA3G was determined according to a previous study, where in a stereoisomer of DPA3G isolated from the stem bark of *Ginkgo biloba*, (1′*R*,3′*S*,5′*R*,8′*S*,2*E*,4*E*)-dihydrophaseic acid 3′-*O*-β-d-glucopyranoside, was reported to have an anti-inflammatory effect at a concentration of 10.7–11.9 μM (approximately 5 μg/mL) [30]. The induction period for the test of cell viability and ALP activity/expression (early osteoblast differentiation marker) of osteoblast-lineage cells was determined according to previous similar studies [50,51]. The cell viability was determined with a WST assay. WST solution (20 μL, 5 mg/mL in phosphate**-**buffered saline) was added to each well, the cells were incubated for another 4 h, and the media were carefully removed. Formazan crystals were dissolved in acidified isopropyl alcohol (40 mM HCl in isopropanol), and their absorbances were measured at 450 nm and 655 nm using a microplate reader (BIO-RAD, Hercules, CA, USA). 

### 3.4. Alkaline Phosphatase (ALP) Activity Assay in Osteoblast Cells 

The preosteoblast MC3T3-E1 cells (3 × 10^3^ cells/well) were incubated in a 96-well plate overnight. Osteoblast differentiation was induced by adding osteogenic medium containing ascorbic acid (50 μg/mL) and β-glycerophosphate (10 mM). Three days after osteoblast differentiation induction, the cells were treated with different concentrations of DPA3G fraction (1, 5, and 10 μg/mL) for 3 days. ALP activity was measured in total cell lysates after homogenization in buffer containing 1 mmol/L of Tris–HCl (pH 8.8), 0.5% Triton X-100, 10 mmol/L of Mg^2+^, and 5 mmol/L of *p*-nitrophenylphosphate as the substrate, and the reaction was stopped using 0.5 N NaOH. The absorbance was read at 405 nm with a microplate reader (BIO-RAD).

### 3.5. Mineralized Nodule Formation in Osteoblast Cells 

MC3T3-E1 cells were incubated in a 48-well plate overnight. The cells were treated with 50 μg/mL of ascorbic acid and 10 mM of β-glycerophosphate for the induction of osteoblast differentiation, with or without treatment with the DPA3G fraction (5 μg/mL), for 21 days, and the medium was changed every 2 or 3 days. The colonies were fixed with 70% ethanol for 10 min at room temperature, rinsed with water, and then stained with 40 mM of Alizarin Red S (Sigma-Aldrich). Positive Alizarin Red S staining was determined with a light microscope. 

### 3.6. Quantitative Reverse-Transcription PCR (qRT-PCR) 

The preosteoblast MC3T3-E1 cells were incubated overnight. Osteoblast differentiation was induced by adding osteogenic medium containing ascorbic acid (50 μg/mL) and β-glycerophosphate (10 mM). Three days after osteoblast differentiation induction, the cells were treated with DPA3G fraction (5 μg/mL) for 3 days. Total RNA was extracted from cultured cells using TRIzol reagent (Invitrogen, Carlsbad, CA, USA) following the manufacturer’s instructions, and RNA quality was assessed by the ratio of absorbance at 260 nm and 280 nm and RT-PCR of *Gapdh* gene. The extracted RNA was subsequently reverse transcribed using a RevertAid™ H Minus First Strand cDNA Synthesis Kit (Fermentas, Hanover, NH, USA), with oligo(dT)_15–18_ as a random primer. All real-time reverse transcription polymerase chain reaction (RT-PCR) measurements were performed using the ABI Prism 7000 Sequence Detection System (Applied Biosystems, Foster City, CA, USA). All PCR amplifications were performed in a total volume of 25 μL containing 150 ng of cDNA using an SYBR Green I qPCR Kit (TaKaRa, Shiga, Japan) according to the manufacturer’s recommendations. The specific primers for osteoblast markers were as follows: 5′-TCC CAC GTT TTC ACA TTC GG-3′ and 5′-GGC CAT CCT ATA TGG TAA CGG G-3′ for mouse *Alpl* (GenBank: NM_007431.3) (117 bp), 5′-TAA AGT GAC AGT GGA CGG TCC C-3′ and 5′-CCT CAG TGA TTT AGG GCG CA-3′ for mouse *Runx2* (GenBank: NM_009820.5) (104 bp), 5′-TAG TGA ACA GAC TCC GGC GCT A-3′ and 5′-ATG GCT TGA AGA CCG CCT ACA-3′ for mouse *Bglap* (GenBank: NM_007541) (135 bp), 5′-CAG CAT CGC TCT GTT CCT GTA-3′ and 5′-CTG CGT TTT CAT GGA GTC TCA-3′ for mouse *Tnfsf11* (GenBank: NM_011613.3) (107 bp), and 5′-TGA CCA CAG TCC ATG CCA TC-3′ and 5′-GAC GGA CAC ATT GGG GGT AG-3′ for mouse *Gapdh* (GenBank: NM_001289726.1) (203 bp). The qRT-PCR conditions were as follows: denaturation at 95 °C for 5 min; amplification with 40 cycles at 95 °C for 5 s, 60 °C for 30 s, and 72 °C for 30 s; and the terminal step for melting at 72 °C to 95 °C for 5 s in each degree. By normalizing to *Gapdh*, the relative quantification of gene expression was performed using the comparative threshold (Ct) method previously described [52]. 

### 3.7. Osteoclastogenesis of Primary Monocytes and Tartrate-Resistant Acid Phosphatase (TRAP) Activity Assay and Staining 

For osteoclastogenesis of primary-cultured monocytes, the isolated monocytes from the bone marrow of mouse femoral bones were cultured in the presence of 30 ng/mL of M-CSF and 50 ng/mL of RANKL (PeproTech) [5,8], with or without DPA3G fraction (5 μg/mL) for 5 days. The cells were fixed in cold 4% paraformaldehyde for 10 min and washed with PBS. The differentiated osteoclast cells from monocytes were measured by a TRAP activity assay and stained using an Acid-Phosphatase Kit (Sigma-Aldrich). TRAP-positive multinucleated cells containing three or more nuclei were counted under a light microscope. The absorbance was measured at 405 nm with a microplate reader (BIO-RAD), and TRAP activity was expressed as the percent of the untreated control.

### 3.8. Co-Culture of MC3T3-E1 Cells and Primary Monocytes

MC3T3-E1 (2 × 10^4^ cells/well) cells were cultured in a 48-well plate overnight. The isolated monocytes (4 × 10^4^ cells/well) from the bone marrow of mouse femoral bones were added in the MC3T3-E1 cells and incubated for 1 day. The MC3T3-E1 cells and primary-cultured monocytes were co-cultured in the osteoblast differentiation-induction media containing ascorbic acid (50 µg/mL) and β-glycerophosphate (10 mM) with or without treatment with the DPA3G fraction (5 μg/mL) for 5 days.

### 3.9. Statistical Analysis

All of the experiments were repeated at least three times with three independent samples, and the results were presented as the means ± standard deviation, as indicated. Statistical analyses were performed using PASW Statistics, version 17.0 (SPSS Inc., Chicago, IL, USA). Statistical significance between the groups was calculated with a Student’s *t*-test. A probability value (*p*) less than 0.05 (*p* < 0.05) was considered statistically significant. Comparisons of multiple groups were done with a one-way analysis of variance (ANOVA), followed by Tukey’s HSD (honest significant difference) post hoc test for correction of multiple comparisons. 

## 4. Conclusions

DPA3G was isolated and identified as one of the candidate bioactive compounds responsible for the bone formation–enhancing effect of LRC extract. DPA3G increased the proliferation, differentiation, and mineralized nodule formation of preosteoblast cells. In co-culture of osteoblast precursor MC3T3-E1 cells and osteoclast precursor monocytes, DPA3G enhanced both osteoblast and osteoclast differentiation, indicating that DPA3G may contribute to enhanced coupling between osteoblasts and osteoclasts in a paracrine fashion, thereby playing a role in the maintenance of normal bone turnover balance. In conclusion, this study demonstrated the role of DPA3G isolated from LRC on enhancing osteoblast differentiation and suggests that DPA3G may be a good candidate for the treatment of osteoporosis.

## Supplementary Materials

Supplementary materials can be accessed at: http://www.mdpi.com/1420-3049/21/9/1260/s1.
