# Supplementary material for: Effects of Dihydrophaseic Acid 3′-O-β-d-Glucopyranoside Isolated from Lycii radicis Cortex on Osteoblast Differentiation
Source: Molecules. 2016 Sep 21;21(9):1260. doi: 10.3390/molecules21091260 (PMC6274582; doi:10.3390/molecules21091260)
Supplement: Supplementary file 1 [file molecules-21-01260-s001.pdf]

Supplementary Materials: Effects of Dihydrophaseic Acid 3'-*O*-β-D-Glucopyranoside Isolated from *Lycii radicis* Cortex on Osteoblast Differentiation

Eunkuk Park, Mun-Chang Kim, Chun Whan Choi, Jeonghyun Kim, Hyun-Seok Jin, Ryunjin Lee, Ji-Won Lee, Jin-Hyok Park, Dam Huh and Seon-Yong Jeong

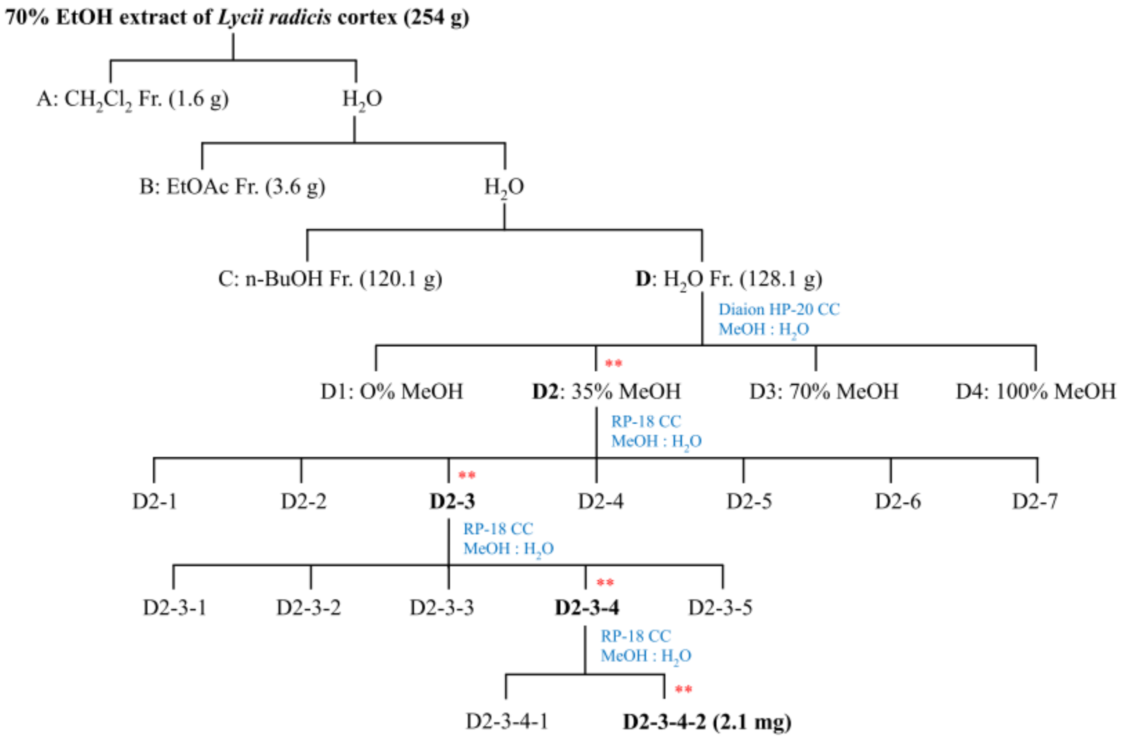

Figure S1. Fractionation and isolation of the bioactive component enhancing osteoblast differentiation from 70% ethanol extract of *Lycii radicis* cortex. Abbreviations: Fr., fraction; CC, column chromatography; EtOH, ethanol; CH_2Cl_2, dichloromethane; EtOAc, ethyl acetate; *n*-BuOH, *n*-butanol; MtOH, methanol. **: Bioactivity-guided fractionation.

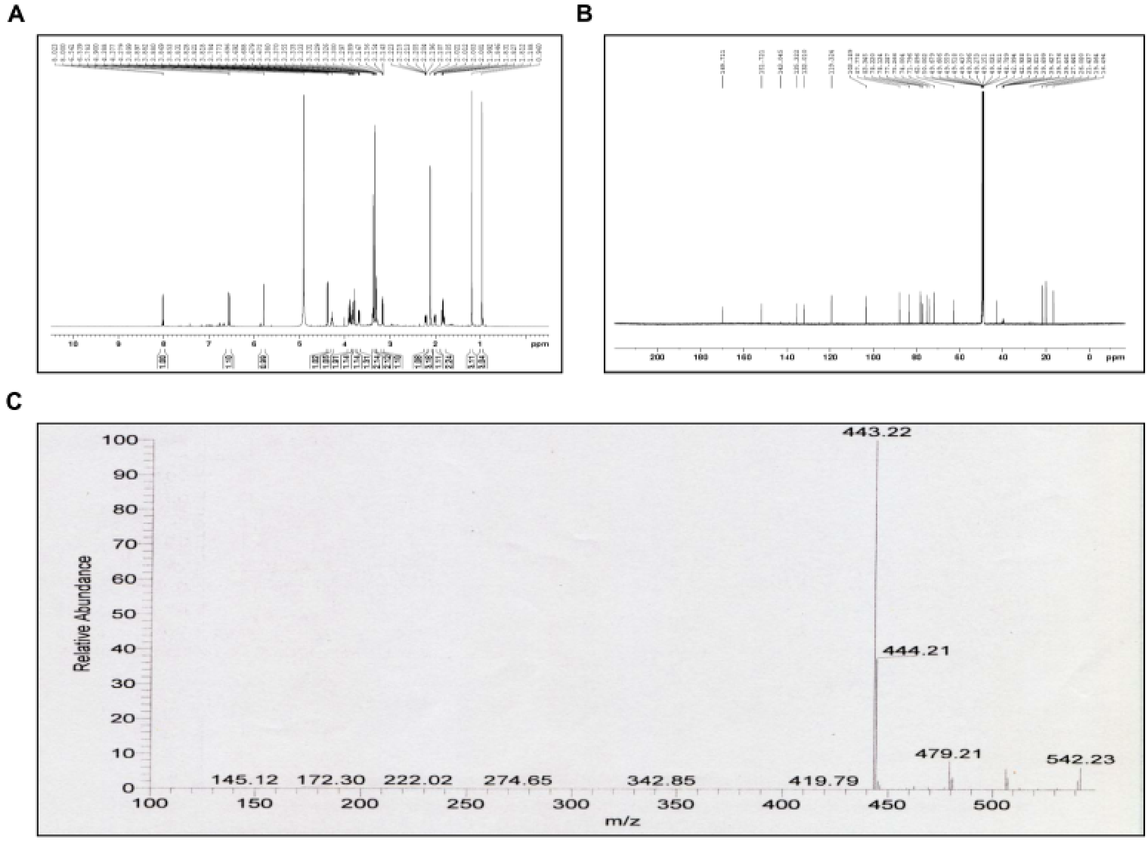

Figure S2. Proton nuclear magnetic resonance (^{1}H-NMR) (A), carbon-13 nuclear magnetic resonance (^{13}C-NMR) (B), and mass spectral (C) analyses of the D2-3-4-2 fraction of Supplementary Figure S1.

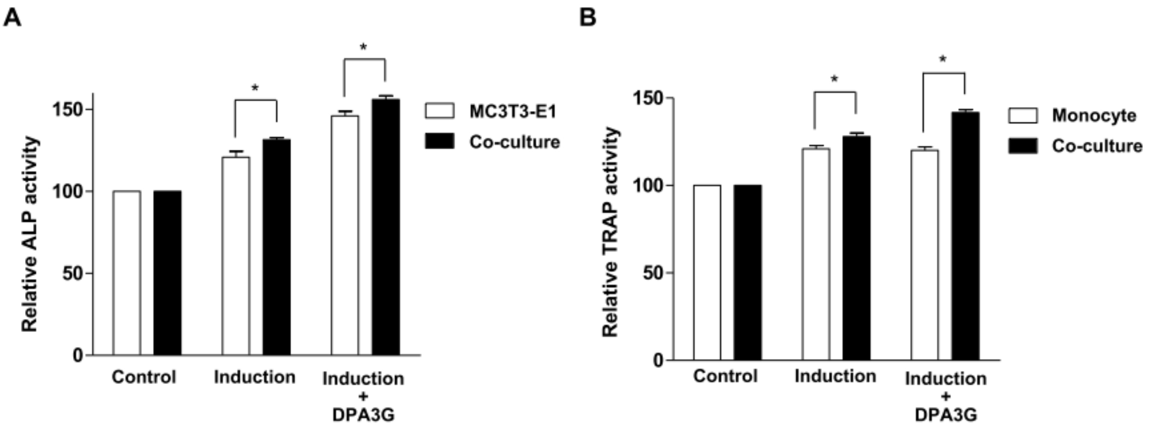

Figure S3. Effect of (1'*R*,2'*S*,5'*R*,8'*S*,2'*Z*,4'*E*)-dihydrophaseic acid 3'-*O*-β-D-glucopyranoside (DPA3G) on osteoblast and osteoclast differentiation in the co-culture of preosteoblasts and primary monocytes. MC3T3-E1 preosteoblasts and primary monocytes were cultured separately or co-cultured. In the separate culture, MC3T3-E1 and primary monocyte cells were treated with osteoblast differentiation reagents (50 μg/mL ascorbic acid and 10 mM β-glycerophosphate) and osteoclast differentiation reagents (30 ng/mL of M-CSF and 50 ng/mL of RANKL), respectively, with DPA3G (Induction+DPA3G) or without (Induction). In co-culture, cells were treated with osteoblast differentiation reagents (50 μg/mL ascorbic acid and 10 mM β-glycerophosphate) with DPA3G (Induction+DPA3G) or without (Induction). After 5 day culture, alkaline phosphatase (ALP) activity (A) and tartrate-resistant acid phosphatase (TRAP) activity (B) were assessed in each cell group. Control: non-induction of osteoblast or osteoclast differentiation. DPA3G: (1'*R*,2'*S*,5'*R*,8'*S*,2'*Z*,4'*E*)-dihydrophaseic acid 3'-*O*-β-D-glucopyranoside. *: $p < 0.05$.

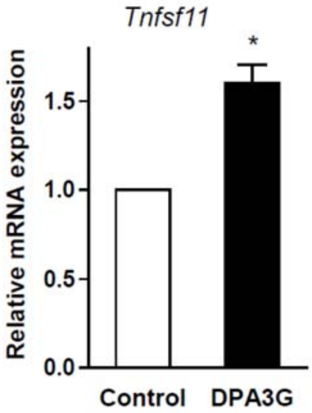

Figure S4. Effects of (1'*R*,2'*S*,5'*R*,8'*S*,2'*Z*,4'*E*)-dihydrophaseic acid 3'-*O*-β-D-glucopyranoside (DPA3G) on *Tnfs11* (LANKL) mRNA expression in the co-culture of preosteoblasts and primary monocytes. MC3T3-E1 preosteoblast and primary monocyte cells were co-cultured for 1 day and then added with 50 μg/ml of ascorbic acid and 10 mM of β-glycerophosphate for induction of osteoblast differentiation. Cells were treated with 5 μg/ml of DPA3G fraction for 5 days and then total RNA of the cells was extracted. The mRNA expression level of *Tnfs11* gene was assessed by quantitative reverse-transcription PCR and then normalized to *Gapdh* mRNA expression. Control: non-DPA3G-treated cells. *: $p < 0.05$ vs. Control.
